# Primary lymph node gastrinoma: a case report and review of the literature

**DOI:** 10.1186/s12957-020-01860-5

**Published:** 2020-04-28

**Authors:** Elisabetta Cavalcanti, Elisa Stasi, Sergio Coletta, Dionigi Lorusso, Caterina Mammone Rinaldi, Raffaele Armentano

**Affiliations:** National Institute of Gastroenterology “S. de Bellis”, Research Hospital Castellana Grotte Bari, Via Turi 27, 70013 Castellana Grotte (Ba), Bari, Italy

**Keywords:** NEN (neuroendocrine neoplasm), Gastrinoma, Primitive lymph node

## Abstract

**Background:**

Gastrinoma is a rare form of neuroendocrine neoplasm. The presence of a primary lymph node localization of gastrinoma is a much debated and controversial topic in the literature, as regards whether these cases represent metastatic disease from an as yet unidentified primary tumor, or the de novo occurrence of a gastrinoma in a lymph node.

**Case presentation:**

We report the case of a 24-year-old male with intense epigastric pain treated at the beginning with high dose proton pump inhibitors. Further workup with CT and subsequent laparotomy revealed a single peripancreatic lymph node. Histological examination highlighted a well-differentiated neuroendocrine tumor.

**Conclusion:**

This case underlines that the primitive lymph node gastrinoma is a distinct nosological entity with a precise location in the context of rare neuroendocrine tumors that should be considered when specific symptoms are associated with the identification of isolated lymph nodes, after excluding any possible primitive locations of neoplastic localization.

## Introduction

Gastrinoma is a rare form of neuroendocrine neoplasm that ectopically secretes gastrin and gives rise to a clinical syndrome of peptic ulcer, first described by Zollinger and Ellison in 1955 [1]. The incidence of gastrinoma is estimated to be in the range of 0.5 to 4 new cases per year per million population [2]. Gastrinomas present either sporadically or associated with multiple endocrine neoplasms type 1 (MEN1), due to an autosomal dominant mutation in the MEN1 gene on chromosome 11q13 [3]. Gastrinomas usually arise in the pancreas and duodenum within the gastrinoma triangle, which defines the confluence of the cystic and common bile duct superiorly, the II and III portion of the duodenum inferiorly and the junction of the neck and body of the pancreas medially [4]; although gastrinomas may occur in both abdominal and extra-abdominal sites [5]. There have been multiple case reports of the existence of primary gastrinomas of the lymph nodes, liver or, more rarely, of the ovary [6, 7]. The presence of a primary lymph node localization of gastrinoma is debated and controversial, as regards whether these cases represent metastatic disease from an as yet unidentified primary tumor, or the de novo occurrence of a gastrinoma in a lymph node. Indeed, some studies report that primary lymph node gastrinomas account for up to 10% of sporadic gastrinomas, while others question this theory, hypothesizing that their presentation represents an undetected microgastrinoma with metastatic lymph node involvement [8, 9]. Herein, we report a case of primitive lymph node gastrinoma, describing the diagnostic workup and operative procedures used to diagnose, localize, and treat the disease at a single institution with much experience of upper biliopancreatic surgery.

## Case report

### Clinical presentation

**Fig. 1:**
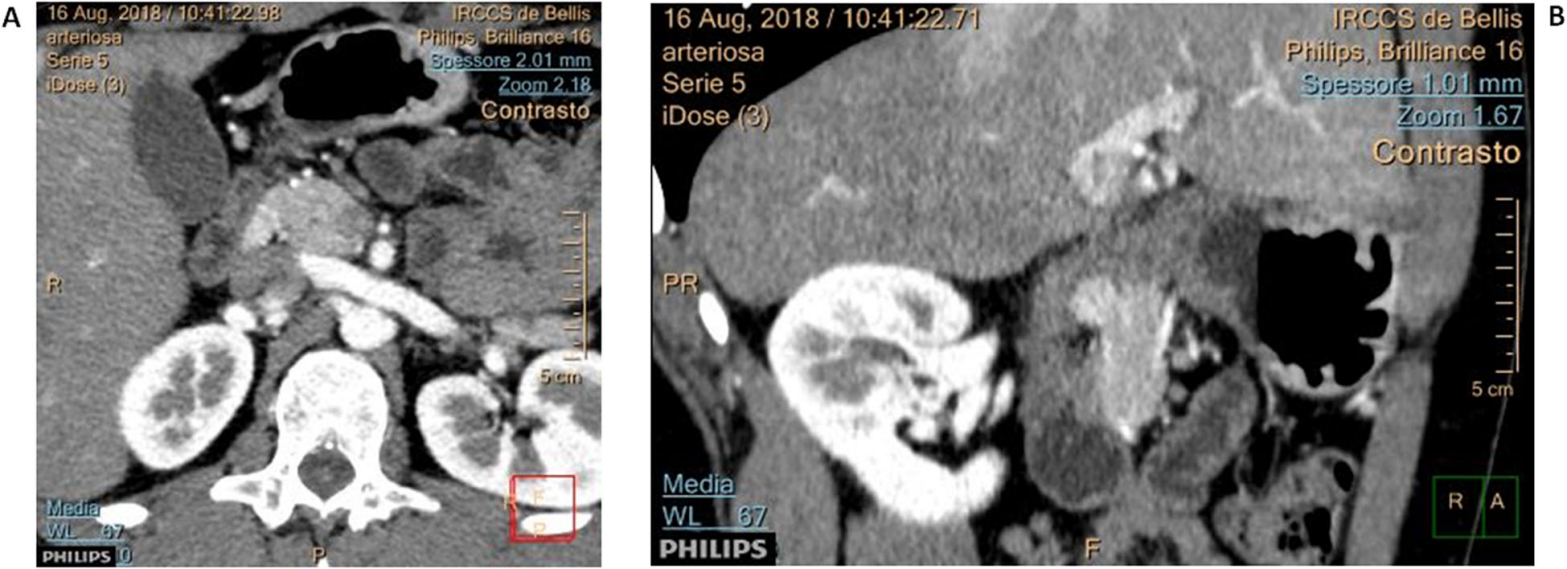
Computed tomography (CT) scan of the abdomen. **a**, **b** Axial sections from abdominal contrast CT demonstrating a 13 mm lesion (arrow) adjacent to the second part of the duodenum, corresponding to an area of abnormal octreotide uptake

A young 24-year-old male presented at our institution for intense epigastric pain, radiating to the right hypochondrium and to the lumbar region, not related to meals nor during nighttime. Informed consent was obtained from the patient studied. All procedures performed were in accordance with the ethical standards of our institutional research committee (IRCCS Giovanni Paolo II, Bari). Previously, for several months, the patient had experienced upper abdominal pain and gained partial benefit from the administration of high dose proton pump inhibitors (PPI). An upper gastrointestinal endoscopy (esophagogastroduodenoscopy, EGD) did not reveal specific erosions or inflammatory lesions of the gastric mucosa, and *Helicobacter pylori* infection was absent. Further workup with abdominal computed tomography (CT) revealed an ovoid lesion measuring 1.3 cm, slightly hypervascularized and exophytic, adjacent to the lateral margin of the head of the pancreas, in the space between the duodenal bulb and the inferior vena cava (Fig. 1). Magnetic resonance imaging (MRI) revealed a single peripancreatic lymph node, suspected to be a metastatic lymph node. No evident injury to the pancreas, biliary tract, stomach, and duodenum was observed. All laboratory test results were within normal limits, except for a significantly elevated fasting gastrin level at 245 pg/ml (normal < 40 pg/ml), chromogranin A level > 2000 ng/ml (normal 10-110 ng/ml), with gastric pH < 2 (off PPI). Therefore, it was not judged necessary to perform the secretin stimulation test, also owing to the gastroscopic exclusion of G-cell hyperplasia. Further laboratory evaluation ruled out a multiple endocrine neoplasia type 1 syndrome (MEN1). Finally, the patient underwent laparotomy, and gave consent to a possible pancreatic-duodenal resection.

### Macroscopic and microscopic examination

**Fig. 2:**
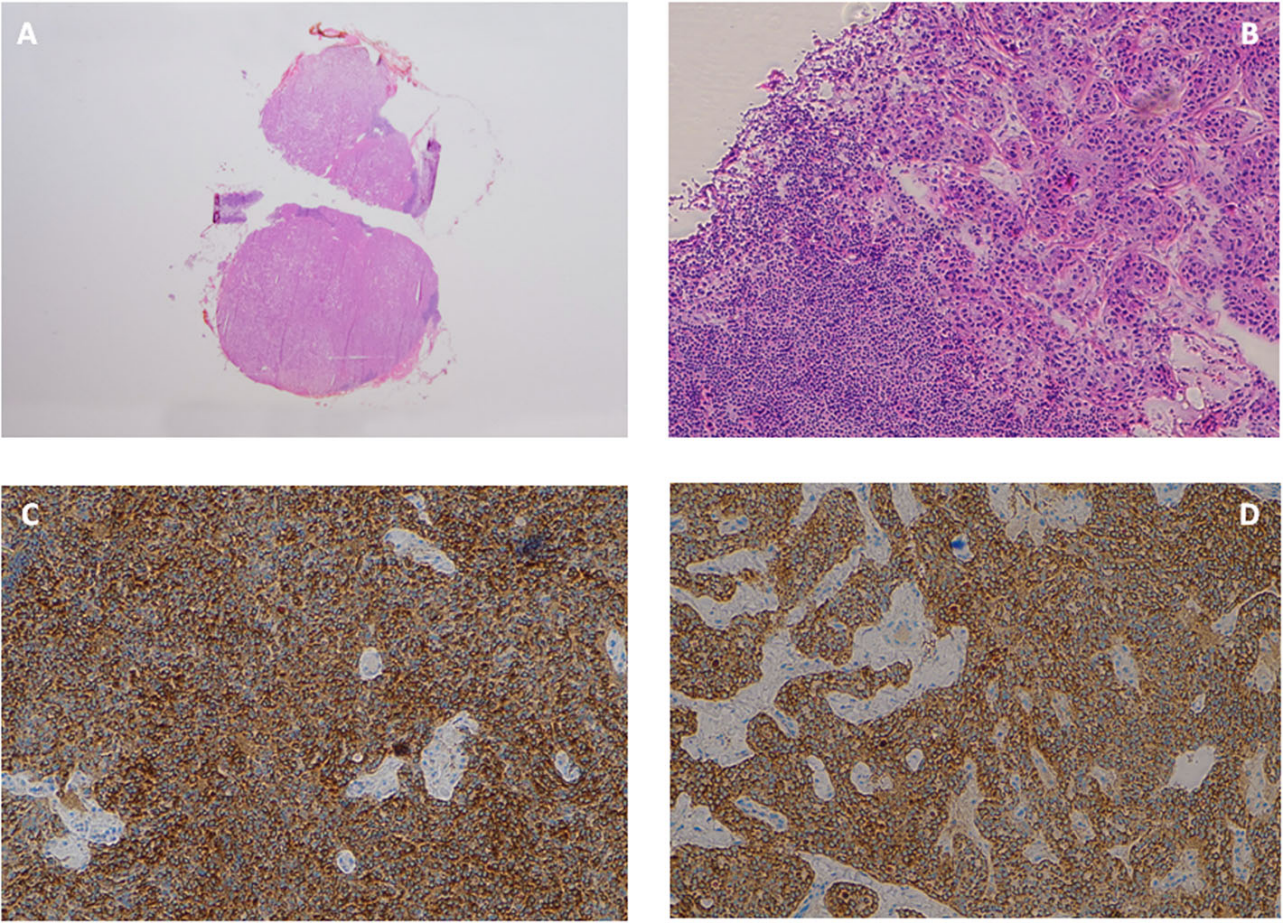
**a** Bisected lymph node almost fully superseded by a neuroendocrine tumor. **b** Histopathology of NET showing subtotal replacement of the lymph node by the NET (HE staining; magnification × 100) immunohistochemistry showed strongly positive staining for synaptophysin (**c**) and weakly positive for gastrin (**d**) (magnification × 100)

Meticulous surgical exploration of the operative field (upper abdomen, including manual duodenal exploration, and sonography of the liver and pancreas) demonstrated the lesion in the pancreatic head, with a diameter of about 2 cm. The nodule, approximately 2 cm in diameter with a smooth, regular surface, was excised and a frozen section submitted to pathology for extemporaneous intraoperative examination. Intraoperative frozen sections analysis disclosed a single lymph node measuring 1.5 cm in maximum diameter, almost completely replaced by a tumor whose appearance was consistent with a low-grade neuroendocrine tumor (NET-G1). After careful exploration of the gastrinoma triangle, no lesion other than the one identified preoperatively was found at surgical exploration and intraoperative ultrasound (IOUS); therefore, further procedures were suspended and the abdomen was closed. The definitive histology and immunohistochemistry analysis on paraffin-embedded sections confirmed a lymph nodule of a well-differentiated neuroendocrine tumor (WD - NET), with less than 3% of cells staining positive for Ki67 (MIB-1) and a number of mitoses < 2 for 10HPF, thereby classified in accordance with 2017 World Health Organization (WHO) classification [10] as a NET G1 (Fig 2a, b). On immunohistochemistry, the lesions showed strongly positive staining for chromogranin and synaptophysin and weakly positive for gastrin (Fig. 2c, d). The diagnosis of a well-differentiated, low-grade, neuroendocrine tumor (NET-G1) was made. The patient’s post-operative course was uneventful and he was discharged home 1 week later. After a post-operative follow-up of 4 months, the patient was asymptomatic, showed significantly improved general conditions and his fasting gastrin and chromogranin A levels were within normal limits.

## Discussion and conclusions

In the literature, the presence of lymph node primary gastrinomas is still controversial due to the uncommoness of the tumor [11]. It is difficult to explain an ectopic localization of gastrinoma. The presence of undetectable micro-lesions of the pancreas and duodenum is often raised to justify the lymph node (LN) localization as a metastatic secondary ties. Only removal of the lymph nodes, in the absence of pancreatic or duodenal resection, however, is curative over time, allowing the gastrin values to return within the normal range. One theory that could explain the existence of lymph node localization, as a consequence of the diffusion of stem cells from the gastrinoma triangle (where 70-90% of such tumors are found) towards the peripancreatic lymph node structures. Consequently, 85% to 90% of gastrinomas lie within the gastrinoma triangle comprising the porta hepatis, duodenal sweep, and pancreatic head [4]. In a prospective study, Norton et al. [12] identified a group of patients who had had only primary lymph node gastrinomas resected and who remained disease-free for up to 20 years, highlighting the existence of primary lymph node gastrinomas. A review of the published cases is presented in Table 1. Most patients are males and affect all age groups. The clinical symptoms are mostly manifest as upper abdominal pain, duodenal ulcers, gastroesophageal reflux disease, weight loss, and diarrhea. In all cases, post-operatively serum gastrin levels have precipitously fallen. The most common localization was the gastrinoma triangle, only in 20% of cases occurred in the peripancreatic lymph nodes. During a median follow-up of 43, 5 (range 4–194) months, all patients have remained asymptomatic. Approximately, 22% (2/9) of gastrinoma cases develop in association with multiple endocrine neoplasia type 1 [20]. In a recent study, Chen Y et al. [19] underlined that primary lymph node gastrinoma was identified in 11/39 cases undergoing consecutively operative gastrinoma resection. Therefore, since no clinical, laboratory, or tumoral characteristic distinguishes these tumors, all ZES patients undergoing surgical resection with curative intent should be subjected to extensive exploration to exclude duodenal, pancreatic, or extrapancreatic tumors and have routine removal of any LNs, especially those in the gastrinoma triangle. In our case, taking into account his younger age at presentation, the patient was screened for MEN1 with negative results. The patient, after only 4 months from surgery, showed complete remission of the epigastric pain symptoms, and return of the gastrinemia values within normal limits, as well as the suspension of proton pump inhibitor drugs. As shown in this case presented, we have good reason to believe that the primitive lymph node gastrinoma is a distinct nosological entity with a precise location in the context of rare neuroendocrine tumors. This entity should be considered when specific symptoms are associated with the identification of isolated lymph nodes after excluding any possible primitive locations of neoplastic localization.

**Table 1:** Literature review of primary lymph node gastrinoma showing clinical features

First Author	Yea	Cases	Age	Gender	Symptoms	Localization gastrinoma	Size	screening MEN1	Gatrin Level	Gastrin Level After surgery	Follow-up period (months)
Olajide 0 [13].	2003	1	77	M	gastric AP	GT	2,5 cm	N	648pg/ml	127pg/ml.	NK
Zhou H [14].	2006	1	39	F	duodenal ulcers , AP	Peripancreatic region	2.4cm	P	1910ng/l	37 ng/l).	144
Mussing K [15]	2009	1	62	F	peptic ulcer disease, WL, AP	GT	2 cm	N	NA	NA	12
Jaenigen B [16].	2009	1	60	M	AP	Retropancreatic region	1 cm	P	1528 ng/L)	120 ng/L.	48
Martin JL [17]	2012	1	42	M	nausea, WL, refluss	GT	9 cm	N	195 pmol/l	32 pmol/l	21
Tenga A[11].	2014	1	57	F	vomiting, watery diarrhea	GT	16 x 19 mm	N	1675 pg/ml	77 pg/ml	NA
Harper S [18].	2015	2	57 76	F M	AP, gastroesophageal reflux	GT	25 x 10 mm 43-mm	N	1053 ng/L 3080 ng/l	69 ng/l 123 ng/l	84 20
Chen Y [19]	2017	11	Mean, 55,2	54,5%, M	AP, gastroesophageal reflux	GT	15 mm (range 3–65)	N	NA	NA	Mean, 59 (11–194)
**Present case**	2020	1	24	M	severe AP	GT	2cm	N	245 pg/ml	3 3 p g / m l	4

*NA* not available, *AP* abdominal pain, *WL* weight loss, *GT* gastrinoma triangle, *N* negative, *P* positive

## Data Availability

All data obtained is available within the manuscript.

## Abbreviations

NEN: Neuroendocrine neoplasia
*ZES*: Zollinger-Ellison syndrome
MEN1: Multiple endocrine neoplasms type 1
PPI: Dose proton pump inhibitors
EGD (esophagogastroduodenoscopy): upper gastrointestinal endoscopy
MRI: Magnetic resonance imaging
CT: Abdominal computed tomography
IOUS: Intraoperative ultrasound
WD – NEN: Well-differentiated neuroendocrine neoplasm
LNs: Lymph nodes
